# Ubiquinol Short-Term Supplementation Prior to Strenuous Exercise Improves Physical Performance and Diminishes Muscle Damage

**DOI:** 10.3390/antiox12061193

**Published:** 2023-05-31

**Authors:** Jorge Moreno-Fernandez, Maria Puche-Juarez, Juan M. Toledano, Ignacio Chirosa, Luis J. Chirosa, Mario Pulido-Moran, Naroa Kajarabille, Isabel M. Guisado, Rafael Guisado, Javier Diaz-Castro, Julio J. Ochoa

**Affiliations:** 1 Department of Physiology, Faculty of Pharmacy, Campus Universitario de Cartuja, University of Granada, 18071 Granada, Spain; jorgemf@ugr.es (J.M.-F.);; 2 Institute of Nutrition and Food Technology “José Mataix Verdú”, University of Granada, 18071 Granada, Spain; 3 Instituto de Investigación Biosanitaria (IBS), 18012 Granada, Spain; 4 Nutrition and Food Sciences Ph.D. Program, University of Granada, 18071 Granada, Spain; 5 Department of Physical Education, University of Granada, 18071 Granada, Spain; 6 Pharmaceutical Laboratory Farmacia Perpetuo Socorro, 18001 Granada, Spain; 7 Department of Preventive Medicine and Public Health, University of the Basque Country (UPV/EHU), 01006 Vitoria, Spain; 8 Nutrition and Obesity Group, Department of Pharmacy and Food Science, Lucio Lascaray Research Institute, University of the Basque Country (UPV/EHU), 01006 Vitoria, Spain; 9 Group of Preventive Activities in the University Field of Health Sciences, Albacete Faculty of Nursing, University of Castilla-La Mancha (Universidad de Castilla-La Mancha/UCLM), 13001 Ciudad Real, Spain; 10 Faculty of Health Sciences, University of Granada, 18071 Granada, Spain

**Keywords:** ubiquinol, muscle damage, intense physical exercise protocol

## Abstract

The benefits of physical exercise on health are diminished when it is non-planned, strenuous, or vigorous, which causes an increase in oxygen consumption and production of free radicals, particularly serious at the muscular level. Ubiquinol could help achieve an antioxidant, anti-inflammatory, and ergogenic effect. The aim of this study is to evaluate whether a supplementation of ubiquinol during a short period could have a positive effect on muscle aggression, physical performance, and fatigue perception in non-elite athletes after high intensity circuit weight training. One hundred healthy and well-trained men, (firemen of the Fire Department of Granada) were enrolled in a placebo-controlled, double-blinded, and randomized study, and separated into two groups: the placebo group (PG, *n* = 50); and the ubiquinol group (UG, *n* = 50), supplemented with an oral dose. Before and after the intervention, data related to the number of repetitions, muscle strength, and perceived exertion, as well as blood samples were collected. An increase was observed in the UG regarding average load and repetitions, revealing an improvement in muscle performance. Ubiquinol supplementation also reduced muscle damage markers, showing a protective effect on muscle fibers. Therefore, this study provides evidence that ubiquinol supplementation improves muscle performance and prevents muscle damage after strenuous exercise in a population of well-trained individuals who are not elite athletes.

## 1. Introduction

The benefits that systematic, regular, moderate, and well-planned physical exercise has on health are well established [1,2]. This is reflected, for instance, by the fact that people with sufficient levels of physical exercise present lower risk of suffering from several chronic diseases [3,4]. These contrasted health benefits are diminished when exercise is unplanned, intense or vigorous, and performed sporadically, without prior high-intensity training, resulting, among other consequences, in increased oxygen consumption in many tissues such as the heart, lungs, and blood [5]. This situation is particularly serious at the muscular level, in which oxygen consumption may be 10 to 15 times higher compared to normal conditions, which would be followed by the consequent increase in the production of free radicals [6,7,8]. Moreover, it has recently been shown that the development of a planned, high-intensity, very low-volume physical exercise protocol can exert a positive immunomodulatory effect, even in cancer patients [9]. However, unplanned and untrained strenuous physical exercise, on the contrary, may induce alterations in various hormonal processes, temporary immunosuppression, and increased susceptibility to infections. It would also produce an over-expression of pro-inflammatory cytokines which, combined with the augmented free radicals, could generate structural damage to muscle cells, which is translated in muscle soreness and swelling, prolonged loss of function, fatigue, and leakage of muscle proteins into circulation [7,10,11,12].

As mentioned above, the implications of free radicals and inflammation in muscle damage and decreased physical performance has been demonstrated, and for this reason, exogenous supplemental antioxidants have received interest as noninvasive useful substances. They are able to decrease muscle damage and improve exercise performance due to their ability to prevent or reduce oxidative stress, lowering specific risks for the pathologic results that strenuous exercise could induce. However, multiple studies on this subject that intend to clarify this relationship have reached mixed outcomes [12,13,14]. In this sense, coenzyme Q would be one of the possible candidates to achieve this antioxidant and ergogenic effect. Coenzyme Q10 (CoQ10) is a key component in the production of energy by the mitochondrial electron transport chain [15,16] even though it is located in all cell membranes, constituting an excellent lipophilic antioxidant [17]. Given its proven capacities, both antioxidant and anti-inflammatory, as well as its role in energy production among other functions, it has become a nutritional supplement administered to seek an improvement in health conditions [18,19].

In the field of sports nutrition, several studies have investigated the effect of CoQ10 on exercise-induced oxidative stress and physical performance, but achieved contradictory results [20,21]. Several studies have shown a positive effect of supplementation with this molecule during exercise, attenuating oxidative stress [22,23], inflammatory signaling, and muscle damage [23,24] and, therefore, improving physical performance [25,26,27,28]. However, other studies show an absence of positive results, and even negative effects related to both muscle oxidative stress and physical performance [16,17,29,30,31]. This wide range of effects may be due to multiple causes, such as the different doses used, the type and intensity of the exercise studied, the physical state of the athletes and the duration of the supplementation [20]. It is also important to highlight that most of the studies carried out to date have used the oxidized form of coenzyme Q10, which has very poor absorption [32].

Currently, it is possible to use the reduced form of coenzyme Q10 (ubiquinol) as a supplement, as it has shown greater bioavailability and therefore an improved ability to increase plasma concentrations at the same dose [33]. In addition, ubiquinol is considered a safe compound, without any side effects, legally authorized, and non-doping [15]. Nevertheless, the optimal plasma level of ubiquinol for athletes is not yet known [34]. Despite this great improvement in the absorption of CoQ10 and considering the advantages of this fact, there are still few studies which involve ubiquinol supplementation during exercise. Moreover, the available studies have some disadvantages, such as a small number of participants or the limited number of parameters determined, leading to controversy among their results [20].

The majority of the available studies in the scientific Literature have been focused on the effect of ubiquinol supplementation during physical exercise in relation to parameters such as oxidative stress, inflammation, muscle damage and, indirectly, physical performance [15,20,28,34,35,36,37,38,39], and even though contradictory data are observed, a positive net effect seems to dominate. Nevertheless, and despite the positive effects discussed above, there is practically no study directly investigating the effect of ubiquinol supplementation on physical performance and fatigue in athletes during high intensity exercise. Consequently, the aim of this study is to evaluate, for the first time, whether a specific supplementation of ubiquinol (200 mg/day) during a short period (2 weeks) could have a positive effect on muscle aggression, as well as on physical performance and fatigue perception, in non-elite athletes after high intensity circuit weight training.

## 2. Materials and Methods

### 2.1. Subjects and Study Design

This study was conducted in a placebo-controlled, double-blinded, and randomized manner. One hundred healthy and well-trained men, (firemen of the Fire Department of the City of Granada) were enrolled in the study. Prior to starting the study, the subjects completed a medical and health history and physical activity questionnaire (IPAQ-SF) [40]. In addition, in order to know the participant’s nutritional status, thus avoiding an important error factor in this type of study, the participants filled out a nutritional questionnaire, which was later evaluated by nutrition software (Nutriber, v1.1.1.5.r5, FUNIBER, Spain). Moreover, body composition assessment was conducted using electrical bioimpedance (EBI) with multifrequency TANITA MC-980MA equipment (Biológica Tecnología Médica S.L., Barcelona, Spain) and Suite Biológica 7.1 software (Version 368). Height was determined using the height contraction measurement method. Participants were instructed to stand still with feet together and heels, buttocks, and upper back in contact with the scale. The parameters examined included weight, basal metabolism, lean mass, fat mass, and total body water. Body mass index (BMI) was calculated by dividing body weight in kilograms by height in meters squared (BMI = kg/m^2^).

All participants were non-smokers, did not use nutritional supplements that might affect the measures, did not present febrile/inflammatory clinical symptoms or chronic diseases, and did not use immunosuppressive or nephrotoxic drugs. They were separated into two groups: the ubiquinol group (UG, *n* = 50) and the placebo group (PG, *n* = 50). For two weeks, the subjects of the ubiquinol group were supplemented with an oral dose of 200 mg (two brown liquid-filled hard gelatin capsules of 100 mg each in the morning, with a glass or water and under fasting conditions). Subjects from the control group took a placebo using the same dose regimen. The capsules, Kaneka QH ubiquinol (Kaneka Corporation, Osaka, Japan), contained 100 mg of ubiquinol solubilized in a matrix of canola oil, diglycerol monooleate, beeswax, and soy lecithin. The placebo capsules contained the same components without ubiquinol and were also supplied by the same company. Weekly supplementation for both groups was prepared through the use of pill boxes that contained a week’s worth of doses. At the end of the week, the participants handed in the empty pillboxes and they were returned refilled with the new doses.

The study was approved by the Commission of Ethics in Human Research of the University of Granada (Ref. 804), and was registered at ClinicalTrials.gov, with registration number NCT01940627. Written informed consent was obtained from each participant, who was free to withdraw from the study at any time in accordance with the Declaration of Helsinki. The flowchart for participant enrolment and dropout is shown in Figure S1.

### 2.2. High Intensity Circuit Weight Training Protocol

Although the physical activity protocol has been previously published [20], we consider that this publication needs to provide a protocol as complete as possible. In the first place, and so as to discriminate the minimum load that every participant can lift in each position of the circuit of the protocol, a pre-training session was carried out a week before the start of the study, based on two parameters: OMNI-RES Scale [41] values of perceived exertion between 6 and 7 and 10 repetitions. After completing the supplementation with ubiquinol for two weeks and before performing the strenuous exercise protocol, the participants performed a two-phase warm-up: a general low-intensity activation phase for 5 min and a specific phase of the exercise protocol to be performed (values 4–5 on the OMNI-RES Scale [41]). The first phase consisted of articular mobility exercises, 5 min low intensity in a cicloergometer and muscle stretching, whereas the second phase corresponded with self-loading exercises and a turn doing the same exercises of the circuit with soft intensity (value 4–5 on the OMNI-RES Scale). Once finished with the warm-up and after a total rest of 5 min, the exercise test was begun.

**Figure 1:**
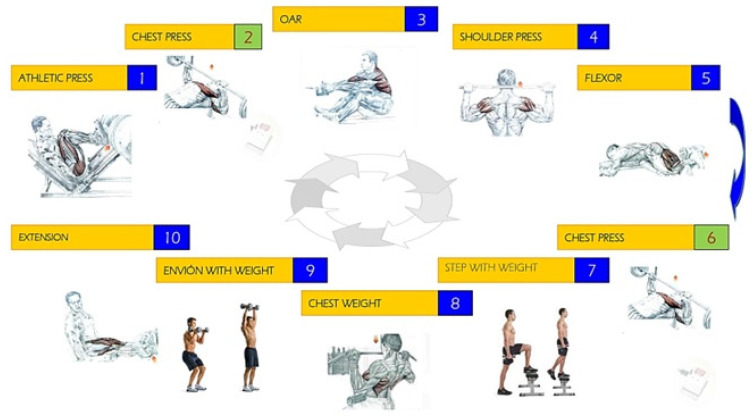
Exercises developed at the high intensity weight training circuit.

Our physical activity protocol (Figure 1) consists of two strenuous physical exercise tests, exercise test 1 (ET1) and exercise test 2 (ET2), with a 24-h rest period between tests. Each test is divided into two sessions, session 1 (S1) and session 2 (S2), with a 5 min rest period between sessions to allow recovery and complete the designed workout [42]. Therefore, the firemen performed two exercise tests with two sets each (S1ET1, S2ET1, S1ET2, and S2ET2). Each of the indicated sessions consists of performing Circuit Weight Training (CWT) composed of 10 bodybuilding exercises: 1, athletic press; 2, chest press in Smith Machine; 3, seated oar; 4, shoulder press; 5, femoral bicep flexion; 6, chest press in Smith Machine; 7, step with weight; 8, surveyor’s pole chest; 9, shove with weight; and 10, quadricep extension. Each exercise was performed for 20 s trying to achieve the maximum number of repetitions and load and always considering a minimum workload corresponding approximately to 60–70% of the Dynamic Maximum Force (DMF or 1RM) [43]. Between each exercise, there was a break of 40 s.

### 2.3. Exercise Tests Data

During the 40 s of rest, an investigator recorded the load and the total number of repetitions in each bodybuilding exercise on an individual record sheet. Additionally, the participants were asked to rate their perceived exertion immediately after the completion of each exercise in the circuit using the OMNI-RES Scale [41] as reference.

### 2.4. Assessment of Muscle Strength

During the performance of the CWT, in bodybuilding exercises 2 and 6, a dynamic system was used to evaluate muscle strength and power. In the case of the bench press in the Smith machine, a linear position transducer device (T-Force System; Ergotech, Murcia, Spain) was used. The system consists of a linear speed transducer cable connected to a computer with specific software that calculates, in real time, different kinematic and kinetic parameters of each repetition. The following variables were measured: maximum speed power (vertical movement of the bar/time (cm/s)), maximum force (ratio of the displaced mass and acceleration manifested throughout movement (Newtons)), and maximum peak power (ratio of the strength and speed recorded during movement (Watts)). The reliability, precision, and validity of this system to measure force, power, and movement velocity has been demonstrated [44].

### 2.5. Blood Sampling

Five blood samples were taken from each participant: before starting ubiquinol supplementation (T1); after completing the ubiquinol supplementation and before starting the first strenuous exercise test (T2); after finishing the first strenuous exercise test (T3); after the end of the 24-h rest and before the second strenuous exercise test (T4); and after finishing the second strenuous exercise protocol (T5).

### 2.6. Biochemical Analyses

Skeletal Muscle Creatine Kinase (CK-MM): An ELISA kit (Cloud-Clone Corp., Houston, TX, USA) was used to measure skeletal muscle injury, using Sandwich enzyme immunoassay for in vitro quantitative measurement in plasma. The assay was performed according to the manufacturer’s protocol. Concentrations were determined by comparison with standards provided by the kit.

Troponin I Type 1, Slow Skeletal (TNNI1): TNNI1 was measured using a commercial kit (Cloud-Clone Corp., Houston, TX, USA). The test principle applied in this kit is Sandwich enzyme immunoassay. Assays were performed according to the manufacturer’s protocols and concentrations were determined by comparison with standards provided by the kit.

Troponin I Type 2, Fast Skeletal (TNNI2): An ELISA kit (USCN Life Sciences, Wuhan, China) was used to determine the concentrations of TNNI2, using Sandwich enzyme immunoassay for in vitro quantitative measurement in plasma following the instructions of the manufacturer. Concentrations were determined by comparison with standards provided by the kit.

Myoglobin (Mb): The quantitative determination of myoglobin in plasma was measured using an ELISA kit (Oxis International Inc., Tampa, FL, USA) following the instructions of the manufacturer, based on the principle of a solid phase enzyme-linked immunosorbent assay.

### 2.7. Statistical Analysis

The data shown in this publication are presented as the mean ± standard error of the mean (SEM). To check if the variables followed the criteria of normality and homogeneity of variance, the Kolmogorov–Smirnoff and Levene tests were used, respectively. The general characteristics of both groups were compared using an unpaired Student’s *t* test. A general linear model of variance for repeated measures with an adjustment by means of Bonferroni’s test was used in order to study the effect of the supplementation and the evolution of time in each studied variable. Bonferroni’s test allowed us to study intra- and inter-subject differences (effect of time in each group and supplementation in each period, respectively). A value of *p* < 0.05 was considered significant. For data analysis, we used the SPSS version 20.0 (SPSS Statistics for Windows, 20.0.0. SPSS, Inc., Chicago, IL, USA).

## 3. Results

No statistically significant differences between groups were found for age, height, weight, body mass index, basal metabolism, percentage and kg of fat mass, lean mass, and total body water at baseline, when the UG and PG were compared (Table 1). Subjects were catalogued as health enhancing physical activity (HEPA active: CATEGORY 3), a high active category, revealing no significant differences between groups. Nutritional intake analyses for the ubiquinol and placebo groups were assessed and previously published [20], revealing that the analysis of a 4-day diet record showed no variations between groups regarding the consumption of macro and micronutrients.

### 3.1. Exercise Test Data

*Load, Repetitions, and Perceived Exertion.* The effect of the intervention on load, repetitions, and perceived exertion in each one of the bodybuilding exercises during the protocol, expressed in mean values of both the UG and PG, are shown in Table S1. 

**Figure 2:**
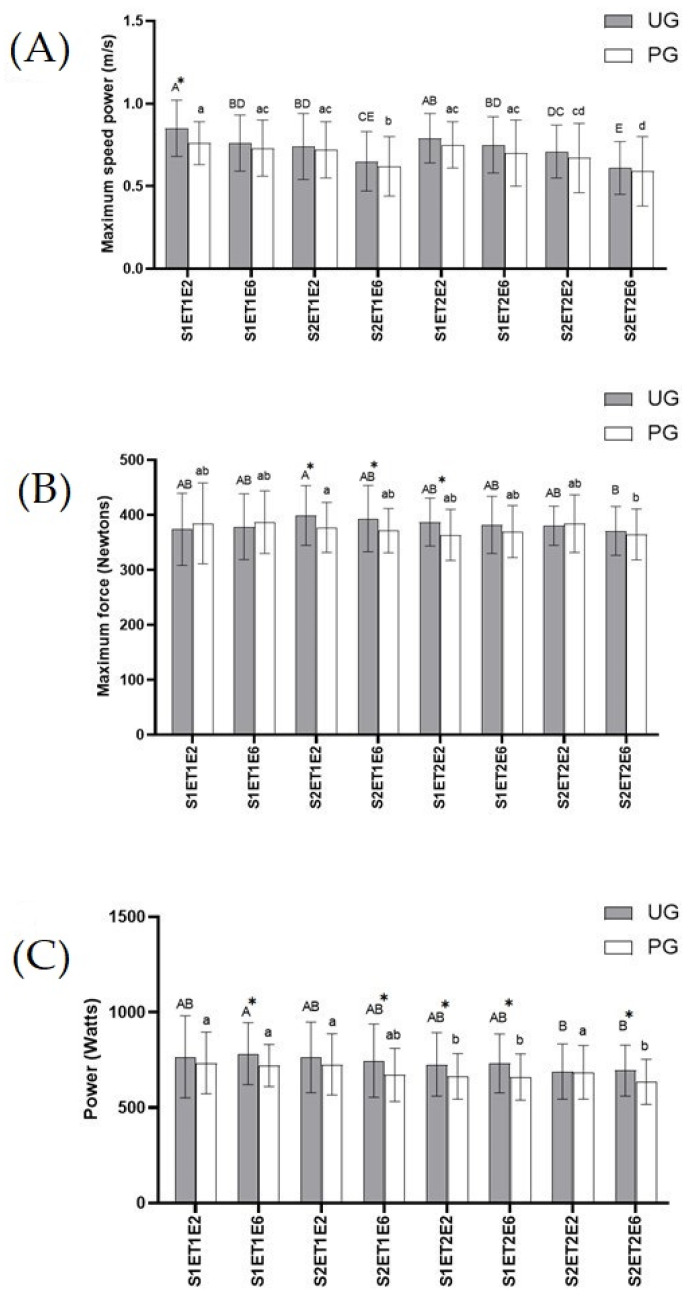
Effects of exercise and ubiquinol supplementation on data obtained in the bench press in Smith machine using a device of linear displacement (T-Force System). * Statically differences between groups. (**A**): Maximum speed power (m/s); (**B**): Maximum Force (Newtons); (**C**): Power (Watt). Values are expressed as means ± SEM. Asterisk means statistically significant differences between groups (*p* < 0.05). Different letters in every group indicates significant differences due to the time (Ubiquinol Group (UG) (A, B, C, D, E); Placebo Group (PG) (a, b, c, d) (*p* < 0.05). S1ET1E2 (Session1—test1-exercise2); S1ET1E6 (Session1—test1-exercise6); S2ET1E2 (Session2—test1-exercise2); S2ET1E6 (Session2—test1-exercise6); S1ET2E2 (Session1—test2-exercise2); S1ET2E6 (Session1—test2-exercise6); S2ET2E2 ((Session2—test2-exercise2); S2ET2E6 (Session2—test2-exercise6).

*Muscle strength*. In exercises 2 and 6 of both tests, maximum speed power, maximum force, and power were measured to assess the muscle strength developed in the bench press in a Smith Machine (Figure 2). According to the results obtained in maximum speed power (Figure 2A), the highest values in both groups were recorded in the second exercise of the first set belonging to ET1. In this point, the UG presented a significant increase (*p* < 0.05) in maximum speed power compared to the PG. No more statistically significant differences were noted among the UG and PG during exercise tests, although results obtained for the UG were always higher than the PG, in all sets and in both bench press exercises (2 and 6) of both exercise tests. The lowest values of maximum speed power for the UG and PG were found in exercise six of the second set included in ET2. This decrease was statistically significant (*p* < 0.05) in the UG compared to the rest of data, except for the results obtained in exercise six of set two in ET1. Meanwhile in the PG the decrease was statically significant (*p* < 0.05) compared to the rest of records obtained, except for data registered in exercise six of set one in ET2.

With regard to maximum force (Figure 2B), there was a significant increase (*p* < 0.05) in the UG compared to the PG in the results recorded for exercises two and six in the second set, belonging to ET1, and in exercise two in the first set of ET2. These values were the highest ones found in the UG. The lowest results for the UG and PG were recorded in exercise six of set two included in ET2, which in the PG was only statistically significant (*p* < 0.05) compared to exercise two of set two in ET1, while in the UG no significant differences were noted in comparison to any other record.

As for power data obtained (Figure 2C), as occurred in maximum speed power, the UG showed higher values than the PG, although these values were only statistically significant (*p* < 0.05) in exercise six of the second set of ET1 and in exercise six in both sets of ET2. No statistical differences were found across the UG. The lowest significant value (*p* < 0.05) in the PG was recorded in exercise six of set two belonging to ET2, compared to the results obtained in exercise two of set one in ET2, and compared to all values obtained in ET1.

### 3.2. Biochemical Analyses

Table 2 shows the effects of exercise and ubiquinol supplementation on muscle damage biochemical parameters. These muscle damage markers included are Creatine Kinase-MM (CK-MM), Troponin I Type 1 (TNNI1), Troponin I Type 2 (TNNI2), and Myoglobin (Mb), respectively. 

With regard to CK-MM, a significant decrease (*p* < 0.05) was found in the UG compared to PG in T5. The highest values of CK-MM were obtained in T5 in both groups, with this increase being statistically significant in the PG (*p* < 0.05) in comparison to values obtained in T1, T2, and T4, while in the UG it was only significant (*p* < 0.05) in comparison to T1.

Regarding TNNI1 levels, a significant decrease (*p* < 0.05) was found in the UG with respect to the PG in T2. However, higher levels were obtained in T3, although this trend changed after T4 and T5, respectively, where TNNI1 levels were lower, though not statistically significant. The highest level in both groups was obtained in T5, which was statistically significant (*p* < 0.05) compared to levels obtained in the remaining data across each group.

As for TNNI2, the highest level in the UG was registered in T5, being statistically significant (*p* < 0.05) compared to T1, T2, and T4. Nevertheless, in the PG, the highest level was found in T3, being significant (*p* < 0.05) compared to the remaining data across groups. A significant decrease (*p* < 0.05) was noted in the UG compared to the PG in T3.

With regard to data obtained in Mb, the highest values were found in T3 and T5 in both groups. A significant decrease (*p* < 0.05) was found in the UG compared to the PG after the supplementation period (T2) and T5. Significant differences (*p* < 0.05) in Mb across groups were found in T3 and T5, each point being compared with the remaining data. 

## 4. Discussion

Oxidative stress and inflammation related to strenuous exercise are two of the key factors that induce muscle damage and decrease muscle performance [7,10,21]. Antioxidant supplementation, which has been postulated as an alternative to prevent or decrease these undesirable effects carried out by reactive oxygen species, could promote an increase in endurance and lead to exercise performance improvements [13,28].

In the present study, we tried to assess the effect of ubiquinol (the reduced form of CoQ10) as a short term supplementation in healthy and well-trained subjects so as to evaluate muscle damage induced by strenuous exercise, together with muscle performance. The exercise protocol performed has previously been reported as a high intensity protocol, with an elevation of lactate and nitric oxide levels, as well as a high oxidative aggression and, therefore, muscle damage [20]. In addition, our ubiquinol supplementation has provided a significant increase in plasma CoQ10 levels, as it has been previously documented [20].

Subject baseline characteristics do not show statistical differences between the UG and PG when it comes to age, height, weight, body mass index, basal metabolism, percentage and kg of fat mass, lean mass, and total body water. Moreover, as we previously published [20], both groups presented similar nutrition, so they constituted two homogeneous groups. As for physical activity, the results obtained from the evaluation of the Short International Physical Activity Questionnaire (IPAQ-short) reveals a high physical activity profile with a total PAscore > 3000 MET 3 min/week, according to the Physical Activity Classification Criteria [40]. 

**Figure 3:**
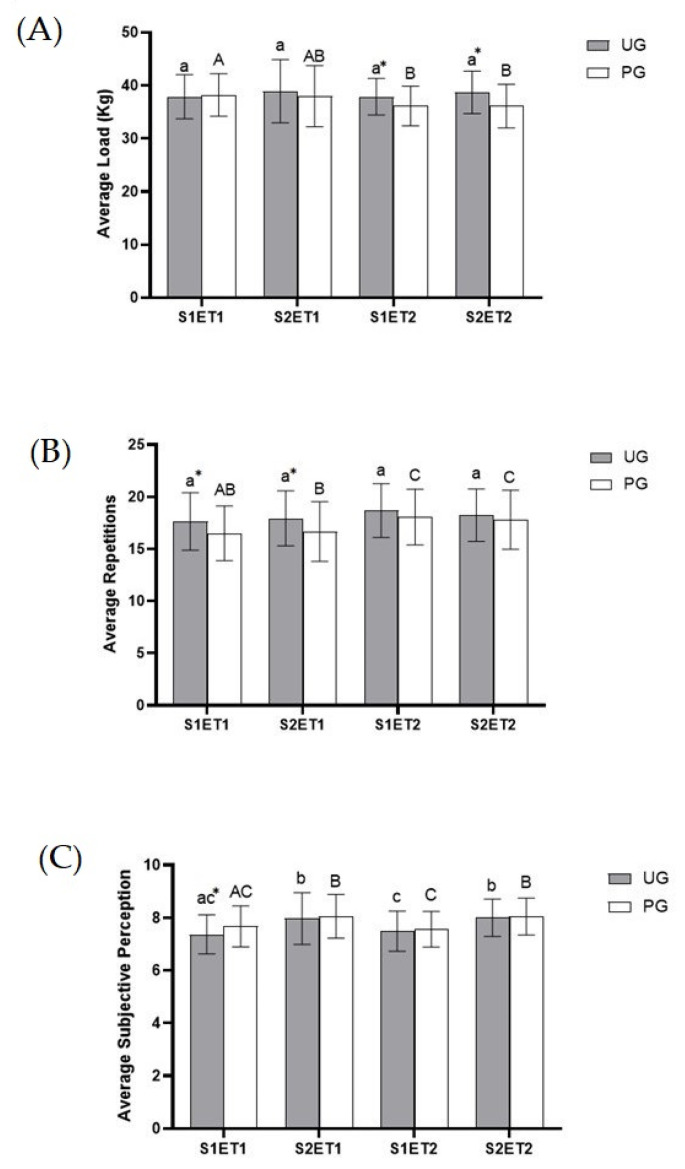
Effect of exercise and ubiquinol supplementation on Average Load (**A**), Average Repetitions (**B**), and Average Perceived Exertion (**C**) obtained from CWT during the exercise protocol. Values are expressed as means ± SEM. An asterisk means statistically significant differences between groups (*p* < 0.05). Different letters in every group indicates significant differences due to the time (Ubiquinol group (UG) (A, B, C); Placebo group (PG) (a, b, c) (*p* < 0.05). S1ET1 (Session 1—Exercise test 1); S2ET1 (Session 2—Exercise test 2); S2ET2 (Session 1—Exercise test 2); S2ET2 (Session 2—Exercise test 2). * Significant differences between groups.

Immediately after the onset of exercise, afferent inputs from contracting skeletal muscles, and in other organs, fine-tuned hemodynamic responses occur to meet the increased metabolic needs produced by exercise. To facilitate oxygen delivery, there is a slight increase in arterial hemoglobin and, in humans, this increase occurs primarily as a result of hemoconcentration due to fluid extravasation. This is why vasodilation occurs in order to increase blood supply and thus oxygen levels, exerting a positive ergogenic effect [45]. Therefore, the higher blood supply could explain some results related to the muscle performance parameters. One objective of this study was to evaluate if ubiquinol supplementation could improve physical performance in healthy trained subjects. Results related to the average load (Figure 3A), as well as to the average repetitions carried out during the sessions (Figure 3B), indicate a positive effect of this intervention. In both ET, the UG delivered a better physical performance; it was observed that the UG lifted more weight in ET2 in comparison to ET1. In this sense, the UG experienced an increased average load and increased repetitions as a general trend—not in all the sets or exercise tests, but in the majority of them—which is in agreement with other studies previously reported by Alf et al. [34]. However, the participants of this study were elite athletes, whereas our study included physically active but non elite athletes, subjects in which this type of high in-tensity activity could cause more damage, fatigue, and less physical performance.

Physical performance is affected by fatigue, and it is important to remark that fatigue has a physical and psychological component [36,46]. Therefore, in this study we tried to analyze this psychological component evaluating the perceived exertions through the OMNI-RES Scale [41]. In most cases, the perceived exertion was similar in both groups. Even so, it must be taken into account that the effort made was not the same in both groups, as the load and repetitions were higher in the UG. Thus, if the values of perceived exertions are adjusted in relation to the number of repetitions in ET2, a reduction in the perceived exertions of 8–10% could be observed in the UG. If the values of perceived exertions are adjusted in relation to the load in ET1, the perceived exertions would be reduced by 6–8% in the supplemented group.

This effect of ubiquinol supplementation on subjective perception is similar to what was observed by Mizuno et al. [26], who demonstrated that oral administration of CoQ10 improved subjective fatigue sensation and physical performance during fatigue-inducing workload trials on a bicycle ergometer. In addition, parameters related to muscle strength and exercise performance presented an increasing trend in the values of maximum speed power, maximum force, and power in the UG in comparison to the PG, although significant differences were noted only in some points. In this sense, there is controversial information regarding CoQ10 supplementation. Some studies noted an improvement in exercise performance in both untrained and trained subjects. Gökbel et al. [27] concluded that 100 mg/day during 8 weeks of CoQ10 supplementation increases exercise performance, especially anaerobic capacity, during repeated bouts of supramaximal exercises in sedentary men. Furthermore, Alf et al. [34] also reported that a supplementation of 300 mg of ubiquinol for 6 weeks enhanced physical performance. 

The possible mechanisms through which our ubiquinol supplementation could improve physical performance and decrease subjective perception may be several. On the one hand, as we previously mentioned, muscle contraction, which acts upon skeletal levers, produces movement. This movement relies on the availability of energy to power it. In vertebrates, movement in general is achieved through the recruitment of motor units in skeletal muscles and the subsequent contraction of muscle fibers, resulting in coordinated limb movement. This energy is provided by adenosine triphosphate (ATP), which can be sourced from high-energy phosphate stores, anaerobic metabolism, or the aerobic production of ATP by mitochondria. These fuels can be supplied to the muscles through the bloodstream in the form of glucose and free fatty acids derived from other tissues and oxygen [45]. Therefore, a higher blood, oxygen, and nutrient supply in the muscle tissue coincides with an increase in nitric oxide (NO) with vasodilator potential at the muscle level and a decrease in oxidative aggression [20]. On the other hand, the improvement could be due to an increase in ATP and creatine phosphate synthesis, which implies anaerobic output. Ubiquinol supplementation could work via a direct increase in muscular Q10 levels, contributing to a rise in ATP synthesis in the mitochondria, and that aerobic energy conversion might be improved by inhibiting ammonia production from AMP [34]. With regard to investigations involving trained subjects, Ylikoski et al. [47] conducted a study with elite cross-country skiers, and concluded that CoQ10 supplementation (90 mg/day during 6 weeks) improved all measured indexes of physical performance (aerobic and anaerobic threshold and VO2max). In this sense, Cooke et al. [25] determined whether an acute single dose of 200 mg and/or chronic (14-days) supplementation of CoQ10 improved anaerobic and aerobic exercise performance, concluding that CoQ10 tended to increase the duration of exercise to exhaustion in healthy untrained and trained individuals.

Previously, and as an indicator of the intensity of the exercise protocol, we have evaluated the lactate levels and its increase during the execution of the exercise (plasma lactate levels ins mmol/L for Placebo Group: T1 = 2.95 ± 0.15 and T5 = 10.45 ± 0.60. For ubiquinol Group: T1 = 2.74 ± 0.14 and T5 = 10.30 ± 0.55) [20]. According to the results obtained, the physical activity performed was strenuous [48], which could lead to muscle damage [39,49].

Muscle damage in exercise could be characterized by the levels of serum markers such as creatine kinase, myoglobin, or troponin (TNNI1 and TNNI2 isoforms) [50]. In relation to CK, the muscle isoform (CK-MM) is one of the most commonly used indirect indicators, as it is an abundant muscle-specific isoenzyme of creatine kinase [51]. This isoform is found in myofibrils, where ATP consumption is high. In this sense, we can observe that there was a trend in CK-MM to progressively increase in the subsequent experimental time points in both groups, revealing the cumulative damage of our strenuous exercise protocol in the muscle fibers, as described in other studies that, however, determined serum CK (mix isoforms) [52]. Nevertheless, ubiquinol supplementation led to a decrease in this parameter in all time points, especially in T5, which could be attributed to the stimulating effect of ubiquinol in mitochondrial biogenesis [53] and the reducing effect of oxidative stress [20]. This would improve ATP synthesis and muscle regeneration and prevent ROS damage, thus reducing the output of secondary metabolites such as CK-MM. 

In the case of Mb, exercise promotes a noteworthy increase after the exercise [54]. As previously described [20], ubiquinol features a muscle-protective effect due to its bioenergetic and antioxidant activity. It is involved in energy production [38], preventing peroxidative damage to membrane phospholipids and reducing free-radical-induced oxidative damage to proteins and mitochondrial DNA. This results in lower peroxidation of the muscle membrane and the subsequent release of Mb after strenuous exercise in the supplemented groups. With regard to TNNI1, both exercise tests increased its levels in both groups, revealing the physical challenge of strenuous exercise for the slow skeletal muscle fibers. These fibers are unable to cope with the oxidative phosphorylation demand to supply ATP for the metabolism of the cells during intense physical exercise. As for Mb, although there is a trend to decrease in all time points, being statistically significant in T3, this reduction can be due to the ergogenic potential of ubiquinol [20], which improves energy supply and metabolic capacities in the muscle fibers avoiding the damage. These results are in agreement with other studies [24,37], where CoQ10 supplementation reduced serum markers of muscle damage indicating that muscular injury was attenuated by the intervention. However, in these studies, the oxidized form of CoQ10 was used, and the participants were elite athletes.

Although this protective effect on muscle damage observed for ubiquinol supplementation has not been described in other studies [31,35], there are important factors to consider. Firstly, the intensity of the exercise; secondly, the training degree of the participants; and mainly, the dose and form of CoQ10 administered. Kizaki et al. [35] carried out the only previous study which used ubiquinol, indicating that the lack of positive results seems to be owing to the low intensity exercise performed, which did not cause the desired muscular damage.

This study features some strengths and limitations that should be taken into account. Regarding the strengths, despite the strong evidence of the detrimental effect of oxidative stress during physical exercise, to date there are very few studies in the scientific Literature evaluating the effect of ubiquinol supplementation during strenuous physical exercise. Furthermore, this study focuses on short-term supplementation to deal with possible one-off oxidative stress, for which the athlete is unprepared, avoiding the detrimental effects of long-term supplementation with antioxidants. Under the heading of limitations, the following should be noted; the study has focused on healthy and well-trained men, but more population groups of athletes could be included, among which elite athletes could be included, with whom to establish a comparison of the possible beneficial effects that the proposed ubiquinol supplementation may have. In addition, different doses of ubiquinol could be tested in order to analyze whether it is necessary to include different intake recommendations depending on the type of athlete. In our study only men are included as healthy and well-treated athletes, because the population group on which the effect of ubiquinol supplementation was evaluated were firefighters, and men predominate in this profession. Therefore, it would be necessary to include women in future studies.

## 5. Conclusions

Our study reveals the effect of a short term ubiquinol supplementation (200 mg/day for 2 weeks) on muscle performance and muscle damage in healthy, moderately trained subjects. In the UG, there is an increase in average load and repetitions and a decrease in perceived exertions as a general trend, revealing an improvement in muscle performance. Ubiquinol supplementation also reduces muscle damage markers, showing a protective effect on muscle fibers after strenuous exercise. 

Therefore, this study firstly provides evidence pointing out that short term ubiquinol supplementation improves muscle performance and prevents muscle damage after strenuous exercise, especially in trained but non-elite athletes, or participants with a high training routine.

## Figures and Tables

**Table 1:** Subjects Baseline Characteristics.

	UG	PG
Age (y)	38.9 ± 1.4	38.2 ± 1.2
Height (cm)	175.4 ± 0.8	174.4 ± 1.2
Weight (Kg)	76.8 ± 1.4	76.3 ± 2.0
Body mass index (Kg/m^2^)	25.0 ± 0.4	25.0 ± 0.5
Basal metabolism (kcal)	1741.8 ± 37.9	1777.8 ± 53.6
Fat mass (%)	18.1 ± 0.9	17.8 ± 1.2
Fat mass (kg)	13.7 ± 0.9	14.2 ± 1.6
Lean mass (kg)	61.6 ± 1.5	62.6 ± 2.0
Total Body water (kg)	45.1 ± 1.1	45.9 ± 1.4

Values are means ± SEM. UG (Ubiquinol group); PG (Placebo group).

**Table 2:** Effect of exercise and ubiquinol supplementation on plasmatic biomarkers of aggression or muscle damage.

PLASMATIC					
	T1	T2	T3	T4	T5
**Creatine Kinase-MM (ng/mL)**					
**UG**	2.02 ± 0.33 ^A^	2.27 ± 0.34 ^AB^	2.67 ± 0.32 ^ABC^	3.06 ± 0.46 ^BC^	3.15 ± 0.31 ^C^*
**PG**	2.01 ± 0.35 ^a^	2.31 ± 0.40 ^ab^	3.20 ± 0.51 ^bc^	3.42 ± 0.36 ^c^	4.00 ± 0.33 ^c^
**Troponin I Type 1 (pg/mL)**					
**UG**	3303.30 ± 203.84 ^A^	3106.79 ± 126.70 ^A^*	4363.31 ± 155.31 ^B^	3396.56 ± 173.22 ^A^	4898.35 ± 228.67 ^C^
**PG**	3448.02 ± 183.10 ^a^	3438.54 ± 130.72 ^a^	4198.44 ± 204.28 ^b^	3426.90 ± 164.11 ^a^	4957.48 ± 321.38 ^c^
**Troponin I Type 2 (pg/mL)**					
**UG**	3328.90 ± 213.11 ^A^	3306.89 ± 201.46 ^A^	3508.95 ± 214.02 ^AB^*	3215.17 ± 241.83 ^A^	3898.65 ± 178.68 ^B^
**PG**	3447.39 ± 205.37 ^a^	3515.20 ± 174.21 ^a^	4217.46 ± 236.69 ^b^	3423.63 ± 255.46 ^a^	4050.33 ± 226.49 ^c^
**Myoglobin (ng/mL)**					
**UG**	24.46 ± 2.30 ^AC^	20.91 ± 1.55 ^A^*	78.74 ± 7.85 ^B^	26.90 ± 2.07 ^C^	80.43 ± 7.55 ^B^*
**PG**	25.09 ± 4.00 ^a^	26.18 ± 2.78 ^a^	92.16 ± 10.21 ^b^	29.43 ± 3.13 ^a^	99.29 ± 8.27 ^b^

* Values are expressed as means ± SEM. An asterisk means statistically significant differences between groups (*p* < 0.05). Different letters in every group indicates significant differences due to the time (Ubiquinol group (UG) (A, B, C); Placebo group (PG) (a, b, c) (*p* < 0.05). T1: before starting ubiquinol supplementation; T2: after completing the ubiquinol supplementation and before starting the first strenuous exercise test; T3: after finishing the first strenuous exercise test; T4: after the end of the 24-h rest and before the second strenuous exercise test; T5: after finishing the second strenuous exercise protocol.

## Data Availability

The raw data supporting the conclusions of this article will be made available by the authors, without undue reservation.

## Supplementary Materials

The following supporting information can be downloaded at: https://www.mdpi.com/article/10.3390/antiox12061193/s1, Figure S1: Flowchart showing participant progress and dropouts in the study. Table S1: Effect of exercise and ubiquinol supplementation on load, repetitions and perceived exertion data obtained from each bodybuilding exercises in CWT during the exercise protocol.
