# Supplementary material for: Ubiquinol Short-Term Supplementation Prior to Strenuous Exercise Improves Physical Performance and Diminishes Muscle Damage
Source: Antioxidants (Basel). 2023 May 31;12(6):1193. doi: 10.3390/antiox12061193 (PMC10295177; doi:10.3390/antiox12061193)
Supplement: Supplementary file 1 [file antioxidants-12-01193-s001.zip › antioxidants-2338948-supplementary.pdf]

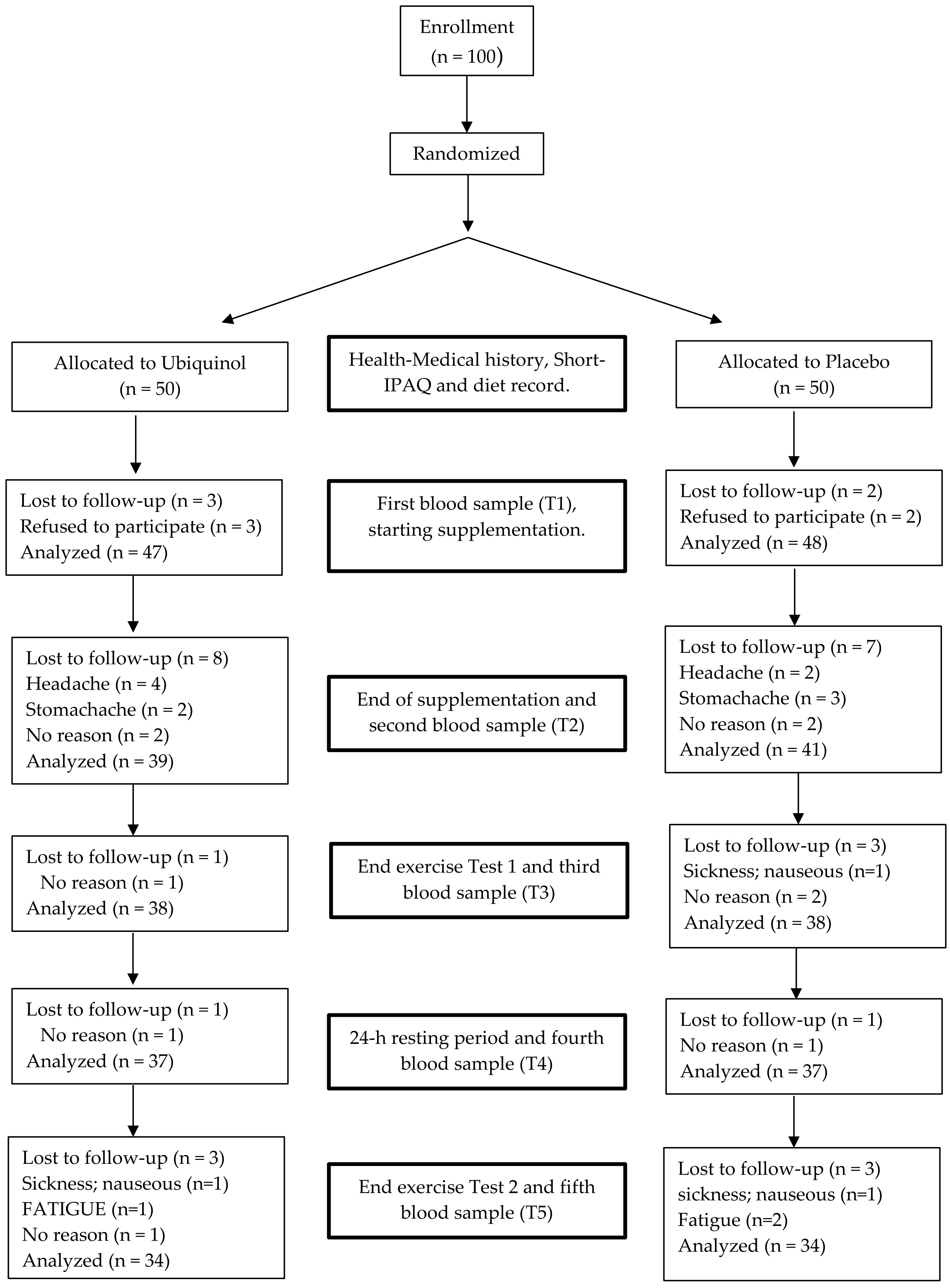
Enrollment
(n = 100)
Randomized
Allocated to Ubiquinol
(n = 50)
Health-Medical history, Short-IPAQ and diet record.
Allocated to Placebo
(n = 50)
Lost to follow-up (n = 3)
Refused to participate (n = 3)
Analyzed (n = 47)
First blood sample (T1), starting supplementation.
Lost to follow-up (n = 2)
Refused to participate (n = 2)
Analyzed (n = 48)
Lost to follow-up (n = 8)
Headache (n = 4)
Stomachache (n = 2)
No reason (n = 2)
Analyzed (n = 39)
End of supplementation and second blood sample (T2)
Lost to follow-up (n = 7)
Headache (n = 2)
Stomachache (n = 3)
No reason (n = 2)
Analyzed (n = 41)
Lost to follow-up (n = 1)
No reason (n = 1)
Analyzed (n = 38)
End exercise Test 1 and third blood sample (T3)
Lost to follow-up (n = 3)
Sickness; nauseous (n=1)
No reason (n = 2)
Analyzed (n = 38)
Lost to follow-up (n = 1)
No reason (n = 1)
Analyzed (n = 37)
24-h resting period and fourth blood sample (T4)
Lost to follow-up (n = 1)
No reason (n = 1)
Analyzed (n = 37)
Lost to follow-up (n = 3)
Sickness; nauseous (n=1)
FATIGUE (n=1)
No reason (n = 1)
Analyzed (n = 34)
End exercise Test 2 and fifth blood sample (T5)
Lost to follow-up (n = 3)
sickness; nauseous (n=1)
Fatigue (n=2)
Analyzed (n = 34)

Figure S1. Flowchart showing participant progress and dropouts in the study.

Table S1: Effect of exercise and ubiquinol supplementation on load, repetitions and perceived exertion data obtained from each bodybuilding exercises in CWT during the exercise protocol.

				bodybuilding exercises									
				**1**	**2**	**3**	**4**	**5**	**6**	**7**	**8**	**9**	**10**
**Load (Kg)**	**ET1**	**S1ET1**	UG	84.4 ± 2.4	43.8 ± 1.8	39.5 ± 1.0	24.0 ± 1.8	36.3 ± 1.2	41.1 ± 1.8	17.3 ± 1.0	40.9 ± 1.2	15.3 ± 0.2	38.1 ± 1.1
			PG	82.2 ± 3.0	43.6 ± 1.3	40.9 ± 1.2	24.4 ± 1.5	36.6 ± 1.3	42.2 ± 1.3	15.9 ± 0.7	38.9 ± 1.4	15.5 ± 0.5	39.5 ± 1.7
		**S2ET1**	UG	85.2 ± 3.7	42.7 ± 1.7	41.6 ± 1.3	23.9 ± 1.7	36.8 ± 1.4	41.6 ± 1.9	17.2 ± 0.9	43.3 ± 1.2	15.3 ± 0.2	38.8 ± 1.3
			PG	79.4 ± 3.6	43.9 ± 1.8	42.1 ± 1.3	22.3 ± 1.5	36.0 ± 1.5	43.3 ± 1.5	16.9 ± 0.9	40.8 ± 1.4	15.8 ± 0.8	39.2 ± 1.7
	**ET2**	**S1ET2**	UG	82.4 ± 3.9	**40.7 ± 0.8***	41.4 ± 1.0	21.6 ± 1.1	37.4 ± 1.1	39.0 ± 1.7	15.5 ± 0.5	**42.6 ± 1.0***	16.2 ± 0.6	**40.9 ± 1.0***
			PG	79.1 ± 3.1	38.2 ± 0.8	41.2 ± 1.5	20.8 ± 1.4	35.8 ± 1.1	39.3 ± 0.9	16.5 ± 0.8	39.3 ± 1.1	16.2 ± 0.7	37.8 ± 1.0
		**S2ET2**	UG	**85.9 ± 2.9***	**40.9 ± 0.8***	**43.8 ± 0.9***	22.4 ± 1.4	36.8 ± 1.0	40.3 ± 1.3	16.0 ± 0.7	**43.4 ± 1.1***	16.4 ± 0.6	**40.7 ± 1.2***
			PG	78.0 ± 2.8	38.5 ± 0.7	39.7 ± 1.0	20.2 ± 1.3	35.3 ± 1.1	39.7 ± 1.0	16.5 ± 0.8	39.8 ± 1.1	16.1 ± 0.6	37.2 ± 1.1
**Repetitions**	**ET1**	**S1ET1**	UG	17.3 ± 3.4	**21.9 ± 0.2***	**19.4 ± 0.4***	**20.5 ± 0.8***	**16.6 ± 0.6***	17.2 ± 0.9	12.4 ± 0.7	16.2 ± 0.5	16.0 ± 0.7	**19.4 ± 0.5***
			PG	17.8 ± 4.4	19.8 ± 0.5	18.0 ± 0.5	18.2 ± 0.6	15.2 ± 0.4	16.8 ± 1.0	11.7 ± 0.7	16.3 ± 0.6	15.0 ± 0.6	17.5 ± 0.4
		**S2ET1**	UG	19.8 ± 0.7	19.4 ± 1.1	19.7 ± 0.6	19.1 ± 1.1	**18.2 ± 0.6***	14.8 ± 1.5	12.5 ± 0.9	16.0 ± 0.6	15.5 ± 0.7	20.1 ± 0.5
			PG	18.7 ± 0.8	19.5 ± 0.9	18.8 ± 0.8	18.7 ± 1.2	16.4 ± 0.5	14.4 ± 1.2	11.1 ± 0.5	16.0 ± 0.6	15.4 ± 0.7	18.5 ± 0.6
	**ET2**	**S1ET2**	UG	20.2 ± 0.8	20.8 ± 0.8	20.4 ± 0.5	21.3 ± 0.8	17.7 ± 0.5	18.8 ± 0.8	13.7 ± 0.6	16.7 ± 0.5	16.7 ± 0.7	20.6 ± 0.5
			PG	19.9 ± 0.9	20.5 ± 0.8	19.9 ± 0.5	21.0 ± 0.9	17.1 ± 0.5	17.5 ± 1.1	13.0 ± 0.7	15.8 ± 0.4	16.3 ± 0.6	19.5 ± 0.5
		**S2ET2**	UG	20.6 ± 0.6	20.5 ± 0.9	20.7 ± 0.5	19.5 ± 1.0	18.6 ± 0.7	15.4 ± 1.1	12.9 ± 0.5	17.0 ± 0.7	16.8 ± 0.6	20.4 ± 0.8
			PG	20.3 ± 0.8	20.2 ± 0.9	20.4 ± 0.5	18.9 ± 0.9	17.6 ± 0.5	14.6 ± 1.2	13.1 ± 0.6	16.3 ± 0.5	16.7 ± 0.5	20.2 ± 0.8
**Perceived Exertion (SCALE OMNI-RES)**	**ET1**	**S1ET1**	UG	7.8 ± 0.7	7.3 ± 0.2	**7.0 ± 0.2***	**7.3 ± 0.2***	**7.0 ± 0.2***	8.3 ± 0.2	7.0 ± 0.2	7.0 ± 0.2	7.6 ± 0.2	7.3 ± 0.2
			PG	7.8 ± 0.8	7.6 ± 0.2	7.6 ± 0.2	7.8 ± 0.2	7.5 ± 0.2	8.2 ± 0.2	7.2 ± 0.2	7.4 ± 0.2	7.9 ± 0.2	7.6 ± 0.2
		**S2ET1**	UG	7.6 ± 0.2	8.0 ± 0.2	**7.9 ± 0.2***	8.3 ± 0.3	**7.3 ± 0.2***	8.6 ± 0.3	7.9 ± 0.2	7.7 ± 0.3	8.1 ± 0.3	7.8 ± 0.2
			PG	7.7 ± 0.2	8.1 ± 0.2	7.4 ± 0.2	8.4 ± 0.2	7.8 ± 0.2	9.0 ± 0.2	7.9 ± 0.2	7.9 ± 0.2	8.5 ± 0.2	8.0 ± 0.2
	**ET2**	**S1ET2**	UG	7.3 ± 0.2	7.8 ± 0.2	**7.0 ± 0.2***	7.7 ± 0.2	**6.9 ± 0.2***	8.2 ± 0.2	7.1 ± 0.2	7.4 ± 0.2	7.8 ± 0.1	7.6 ± 0.2
			PG	7.5 ± 0.2	7.6 ± 0.2	7.4 ± 0.1	7.6 ± 0.2	7.4 ± 0.2	8.3 ± 0.2	7.4 ± 0.2	7.4 ± 0.2	7.7 ± 0.1	7.5 ± 0.2
		**S2ET2**	UG	7.7 ± 0.2	8.1 ± 0.2	7.5 ± 0.2	8.3 ± 0.2	7.4 ± 0.1	8.8 ± 0.2	7.9 ± 0.2	8.0 ± 0.2	8.4 ± 0.2	7.9 ± 0.2
			PG	7.9 ± 0.2	9.9 ± 1.2	7.8 ± 0.2	8.2 ± 0.2	7.5 ± 0.1	9.0 ± 0.2	7.8 ± 0.2	7.9 ± 0.2	8.2 ± 0.2	8.1 ± 0.1

*Values are expressed as means ± SEM. Asterisk means statistically significant differences between groups ($p<0.05$). ET1: exercise test 1; ET2: exercise test 2; S1ET1: Session 1 - Test 1; S2ET1: Session 2 – Test 1; S1ET2: Session 1 – Test 2; S2ET2: Session 2 – Test 2; PG: placebo group; UG: ubiquinol group.

With regard to load, no statistically significant differences were observed in the exercises of S1ET1 and S2ET1. However, differences were found in UG for exercises 2, 8 and 10 during S1ET2, and for the exercises 1, 2, 3, 8 and 10 during S2ET2. When it comes to repetitions, statistically significant differences are not found in the ET2, but during ET1. Specifically, exercises 2, 3, 4, 5 and 10 reported differences between groups in S1ET1, with a higher amount of repetitions performed by UG. In S2ET1, these differences were only observed in exercise 5. As for perceived exertion, differences were reported in both ET1 and ET2. On the one hand, ET1 showed significant differences for exercises 3,4 and 5 in S1ET1, and for exercises 3 and 5 in S2ET1. On the other hand, ET2 only reported differences for exercises 3 and 5 in S1ET2. In all these cases, less perceived exertion was observed in UG compared to PG. Values are expressed as means ± SEM. Asterisk means statistically significant differences between groups ($p<0.05$). ET1: exercise test 1; ET2: exercise test 2; S1ET1: Session 1 - Test 1; S2ET1: Session 2 – Test 1; S1ET2: Session 1 – Test 2; S2ET2: Session 2 – Test 2. UG (Ubiquinol group); PG (Placebo group).
